# Lipids signature shift in *Zea Mays* L. resistant and susceptible inbred lines in response to *Fusarium verticillioides*

**DOI:** 10.1186/s12870-025-07176-4

**Published:** 2025-08-26

**Authors:** Laura Carbonell-Rozas, Noemi Gesteiro, Laura Righetti, Rogelio Santiago, Ana Butrón, Chiara Dall’Asta

**Affiliations:** 1 Department of Food and Drug, University of Parma, Parma, Italy; 2 Laboratory of Organic Chemistry, Wageningen University, Wageningen, WE 6708 The Netherlands; 3 Misión Biológica de Galicia, Sede de Pontevedra (CSIC), Pazo de Salcedo, Carballeira 8, Salcedo , Pontevedra, 36143 Spain; 4 Department of Chemistry and Physics, Research Centre for Mediterranean Intensive Agrosystems and Agrifood Biotechnology (CIAIMBITAL), Agrifood Campus of International Excellence (ceiA3), University of Almeria, Almeria, E- 04120 Spain

**Keywords:** Fusarium ear rot, Plant pathogen interaction, Lipidomics, Mycotoxins, Plant resistance

## Abstract

**Background:**

Fusarium Ear Rot is one of the major diseases affecting maize worldwide, causing decreases in yield and fumonisins accumulation in crops. In this framework, identifying resistance traits in plants is of great interest for breeding programs. To delve deeperr into the role of lipids on resistance to Fusarium Ear Rot, a lipidomic study has been performed using resistant and susceptible maize recombinant inbred lines.

**Results:**

Samples at 10 days after infection underwent untargeted UHPLC-TWIM-HRMS analysis, leading to the putative annotation of 182 compounds significantly over- or under-accumulated in resistant inbred lines. Significant compounds were further investigated to better understand their biological role. Besides the involvement of well-described lipid classes such as oxylipins and phospholipids, this study pinpointed the differential accumulation of phytoceramides and Amadori-glycated glycerophosphoethanolamines in resistant lines.

**Conclusions:**

Taken altogether, our data demonstrated the complex interactions occurring at lipidome levels during plant-pathogen interaction.

**Supplementary Information:**

The online version contains supplementary material available at 10.1186/s12870-025-07176-4.

## Background

Maize (*Zea mays* L.) is one of the most important cereal crops globally, and a critical component of animal feed and industrial products. Maize production is significantly threatened by various fungal pathogens, among which *Fusarium verticillioides* is of utmost relevance due to its prevalence, especially under climate change conditions [1, 2].

Besides causing ear rot and stalk rot in maize, leading thus to substantial yield losses, *F. verticillioides* infection can cause a strong accumulation of fumonisins (FBs) in infected crops. Due to the severe health risks posed by FBs to humans and animals, including carcinogenic effects, the management of *F. verticillioides* infection is a priority in maize cultivation [3].

The resistance of maize to *F. verticillioides* is a complex trait involving multiple low-effect genes and biochemical pathways [4–7]. Traditional breeding methods have focused on selecting resistant varieties, but the underlying molecular mechanisms remain incompletely understood. Recent advances in omics science have shed light on the intricate network of genes and signalling pathways involved in maize resistance to this pathogen [5, 8]. Among these, lipid signalling and metabolism have emerged as critical components of the plant’s defence arsenal [9–11].

Lipids play diverse roles in plant defence, acting as structural components of cell membranes, signalling molecules, and precursors for bioactive compounds. In the context of maize resistance to *F. verticillioides*, several classes of lipids, including phospholipids, sphingolipids [8, 12], and oxylipins, have been recently reported as implicated in the defence response [10, 13]. Oxylipins, derived from the oxidation of polyunsaturated fatty acids (FA), have often been described as the front-line signalling compounds upon fungal infection [3, 12, 13]. In addition, the interplay between lipid signalling, hormonal regulation, and secondary metabolite production further underscores the complexity of maize resistance to *F. verticillioides*. For instance, the biosynthesis of the hormone jasmonic acid involves the conversion of linolenic acid to 12-oxo-phytodienoic acid (12-OPDA), an oxylipin intermediate [14, 15].

Therefore, an untargeted metabolome study was implemented to find, in developing maize kernels, markers for resistance to Fusarium Ear Rot (FER) and kernel contamination with fumonisins, unbiased by genetic background differences [16]. Specifically, the study showed that differences for membrane lipid homeostasis could play a determinant role on differences for resistance between recombinant inbred lines (RILs) obtained from the same cross; the effect being more important at 10 days after inoculation (dai) with *F. verticillioides* (24 days after pollination) than at 3 dai (17 days after pollination). Based on bulk-segregant transcriptomic data using the same RILs, Cao et al. [17] suggested that mobilization of lipids from oil bodies to phytoalexin synthesis could be behind the resistance, but the untargeted metabolic approach showed that lipid re-modelling could be rather redirected toward phosphatidylcholine accumulation. Therefore, in the current paper, to deepen into the role that lipids could have on resistance to FER, a bulk-segregant lipidomic study has been performed using kernel bulks of resistant and susceptible RILs collected at 10 dai.

## Methods

### Chemicals and reagents

The solvents used for sample extraction and chromatographic separation such as methanol and 2-propanol were HPLC-grade and were purchased from Merck (Darmstadt, Germany). Formic acid, and ammonium formate were supplied by Sigma-Aldrich (St. Louis, MO, USA). Water was purified by Milli-Q purification system (Millipore, Bedford, MA, USA).

The lipid standards including mono-glycerols (1-monooleoyl-rac-glycerol (monoolein), 3-monopalmytoyl-sn-glycerol (monopalmitin), 3-monostearoylsn-glycerol (monostearin), 1-linoleoyl-rac-glycerol, 1-monolinolenoyl-rac-glycerol, glyceryl arachidate, 1-monopalmitoleoyl-rac-glycerol), di-glycerols (mix of 1,2-dipalmitoyl-rac-glycerol (1,2-dipalmitin), 1,3-dipalmitoyl-racglycerol(1,3-dipalmitin) isomers, 1,3-distearoyl-racglycerol(1,3-distearin), 1,3-dilinoleoyl-rac-glycerol), and triacylglycerols (glyceryl trioleate, glyceryl trilinoleate and glyceryl tristearate) were purchased from Nu-Check Prep, INC (Elysian, MN, 56028 USA). Individual lipid standards were prepared at 10 mg/L concentration and reconstructed in 2-propanol: methanol: water mixture (65:30:5, *v/v/v*). A 100 µg/L dilution of the lipid mixture was then injected in random sequence during the analysis.

### Sample collection and preparation

As explained in Cao et al. [16], eight RILs of maize were selected from a set of 144 RILs derived from the cross between the susceptible European flint inbred line EP42 and the resistant American dent inbred line A637. The four resistant RILs (RES) had a total fumonisin content (a sum of FB1, FB2 and FB3) in the range 10–15 µg/g and values of 2 for FER, based on a 7-point scale (1 = no visible disease symptoms, 2 = 1–3%, 3 = 4–10%, 4 = 11–25%, 5 = 26–50%, 6 = 51–75%, and 7 = 76–100% of kernels showing visible symptoms of infection) developed by Reid and Zhu (2005). In contrast, the four susceptible RILs (SUS) had total fumonisin content between 55 and 75 µg/g FER score of 4 [17]. Although the difference in FER scores may seem moderate, it reflects a biologically relevant increase in visible infection. Moreover, resistance in this context also includes the plant’s ability to limit fumonisin accumulation, which is not always directly proportional to visible symptoms*.*

Lipidomic analyses were performed using plant material from the 2018 field trial described in Cao et al. [17].The three ears collected from each RIL were assigned to three different RIL mixtures that were freeze-dried and milled until getting a fine powder, resulting in three mixtures of the four resistant RILs and three mixtures of the four susceptible RILs. Then, three different powder samples were taken from each mixture, resulting in nine replicates for each bulk of RILs (RES, resistant or SUS, susceptible).

### Sample extraction

100 mg of fine-grinded maize sample were extracted using a scaled-down solid-liquid extraction based on a previously extraction procedure reported by Rubert et al. [18]. The sample was weighted in a 2 mL Eppendorf tube and 1 mL of a cold mixture composed by dichloromethane: methanol (50:50, *v/v*) were added as extraction solvent. The mixture was vortexed for 15 min at 240 s/min and centrifuged at 14.000 rpm and 4 °C for 10 min. Afterwards, 200 µL of the upper phase was transferred to a glass vial and dried under a gentle nitrogen current. Finally, the sample was reconstituted in 1 mL of 2-propanol: methanol: water mixture (65:30:5, *v/v/v*) before the injection into the UHPLC-TWIMS-QTOF system.

### Untargeted UHPLC-TWIMS-QTOF lipidomics analysis

Lipidomics analysis was performed using an ACQUITY I-Class UPLC separation system coupled to a Vion IMS QTOF mass spectrometer (Waters, Wilmslow, UK) equipped with an electrospray ionization (ESI) interface. Chromatographic separation and MS conditions were set as previously described [19]. Briefly, an injection volume of 2 µL of the extract was injected and chromatographically separated using a reversed-phase C18 BEH ACQUITY column (2.1 × 100 mm, 1.7 μm particle size) (Waters, Milford, MA, USA) and kept at 60 °C. The mobile phase consisted of 5 mM ammonium formate in Milli-Q water/methanol (95:5, *v/v*) (solvent A) and 5 mM ammonium formate in isopropanol/methanol/Milli-Q water (65:30:5, *v/v*) (solvent B), both acidified with 0.1% formic acid. The elution was performed in gradient mode as follows: 0.0 min (10% solvent B; 0.40 mL/min) to 1.0 min (50% solvent B; 0.40 mL/min), subsequently 1–5 min (80% solvent B; 0.40 mL/min), and 11.0 min (100% solvent B; 0.50 mL/min). After a 4.5 min isocratic step, the system was re-equilibrated to initial conditions for 2.5 min (10% solvent B; 0.4 mL/min). During the analysis, the autosampler temperature was maintained at 10 °C.

Regarding MS conditions, analyses were performed in positive and negative electrospray ionization modes, acquiring continuum data in the range of 50-1200 *m/z* with a scan time of 0.15 s. The capillary voltage was set at 2.5 kV, the source and desolvation temperatures were 120 °C and 500 °C, respectively, while the desolvation gas flow was 1000 L/h. The TOF analyzer was operated in sensitivity mode. Data were acquired using High Definition MS^E^ (HDMS^E^), which is a data-independent approach (DIA) coupled with ion mobility. Low-energy scan (CE 6 V) and a high-energy scan (CE ramp 20–40 V) were alternatively acquired during the run. The optimized ion mobility settings were as follows: nitrogen flow rate, 90 mL min^−1^ (3.2 mbar); wave velocity, 650 m s^−1^; and wave height, 40 V.

TOF and CCS calibrations were performed for both positive- and negative-ion modes using a Major Mix IMS/ToF Calibration Kit (Waters, Wilmslow, UK). In the case of the positive-ion mode, the calibration covered the *m/z* range between 152 and 1922 Da and a CCS range from 130.4 to 372.6 Å^2^, while for the negative-ion mode, the *m/z* and CCS ranges were between 150.1 and 1965.9 Da and 131.5-367.2, respectively. LockSpray containing Leucine-Enkephalin ([M + H]^+^ *m/z* 556.2766) at a concentration of 200 pg/µL and infusion rate 15 µL/min was used for real-time mass correction (acquisition every 2.5 min, 3 scans to average).

To address overall process variability, experimental replicates, quality control (QC) samples and a mix of lipid standards were injected throughout the sample list. The QC sample, prepared by pooling an aliquot of the extract from each sample, was injected at the beginning of the sequence and every 10 sample injections. The sample-injection order was randomized to avoid any possible time-dependent changes during UHPLC-TWIMS-QTOF analysis, which could result in false clustering. The reference mixture was injected at the beginning of the sequence and every 10 sample injections to verify retention times as well as *m/z* stability over time. In addition, each set of samples was preceded by three blank controls to avoid carryover or cross-contamination of the sets. Thus, blanks of Milli-Q water, MeOH, and the resulting solvent after applying the extraction procedure without sample were injected.

### Data processing and statistical analysis

Data processing and compound identification were conducted using Progenesis QI Informatics (Version 4.20, Waters Corporation). Each UHPLC-TWIM-HRMS run was imported as an ion-intensity map, including *m/z*, CCS, and retention time, which were then aligned in the retention-time direction (0–15 min). From the aligned runs, an aggregate run representing the compounds in all samples was used for peak picking. This aggregate was then compared with all runs, so that the same ions were detected in every run. Isotope and adduct deconvolution were applied to reduce the number of features detected.

The dataset containing aligned features was exported into MetaboAnalyst 6.0 [20], log-transformed, and Pareto-scaled before evaluating the quality of the unsupervised and supervised models. Principal component analysis (PCA) was performed to assess natural sample grouping. Furthermore, a supervised method, the Orthogonal Partial Least Squares Discriminant Analysis (OPLS-DA), was performed to separate components related to class differences from orthogonal (unrelated) components, thereby facilitating a more straightforward interpretation of the results. From the analysis of the variance (ANOVA), significant features were selected, retaining those presenting Benjamini–Hochberg false discovery rate (FDR) adjusted p-value (q-value) < 0.01 (see Supplementary Material, Figure **S1** for more details).

The resulting significant features were subjected to identification. Metabolites were annotated by publicly available database searches, including PubChem and LipidMaps, as well as by fragmentation patterns for lipids, using Progenesis QI. Based on the strategy applied, identification was carried out according to “level III” (putatively characterized compounds) and, when possible, “level II”, (putatively identified compounds), corresponding to compounds identified by UHPLC-HRMS/MS and matched to spectra from databases and the literature, as set out by the Metabolomic Standard Initiative [21].

Quantitative Enrichment Analysis was carried out using MetaboAnalyst v6.0 [20], while the data was further visualised and plotted using the SRplot online platform [22]. Systematic changes in lipid pathways were visualised using the BioPAN web-based tool [23].

## Results and discussion

For the lipidomics analysis, samples were analysed by UHPLC-Q-TOF-MS under a fully untargeted approach using the same extraction and instrumental conditions as reported in our previous works to allow data comparison [10, 11].As a preliminary step, raw data acquired in both positive and negative ionization modes underwent PCA to detect sample clustering within the measured data and to gain an overview of the overall trends, including the identification of potential outliers (Figure**S2**,Supplementary Information). The QCs were tightly clustered close to the plot center across the entire sequence, suggesting a high quality of data acquisition.

After a quality assessment, the data were filtered by choosing entities presenting FDR adjusted p-values (q-value) below 0.01 and coefficients of variation below 30%, resulting in a reduced dataset of 1,358 and 898 features in ESI + and ESI- respectively.

Filtered features were then putatively identified based on accurate mass, isotopic pattern, and MS/MS fragmentation, using a range of consolidated databases, i.e., HMDB, MassBank, and the LIPIDMAPS. Mass accuracy was set to 5 ppm as acceptance criteria, and the Progenesis fragmentation score was set above 60%. This led to the putative annotation of 182 discriminative markers, among them 136 in ESI + and 46 in ESI- (see Supplementary Information, Table **S1**,for details).

**Fig. 1:**
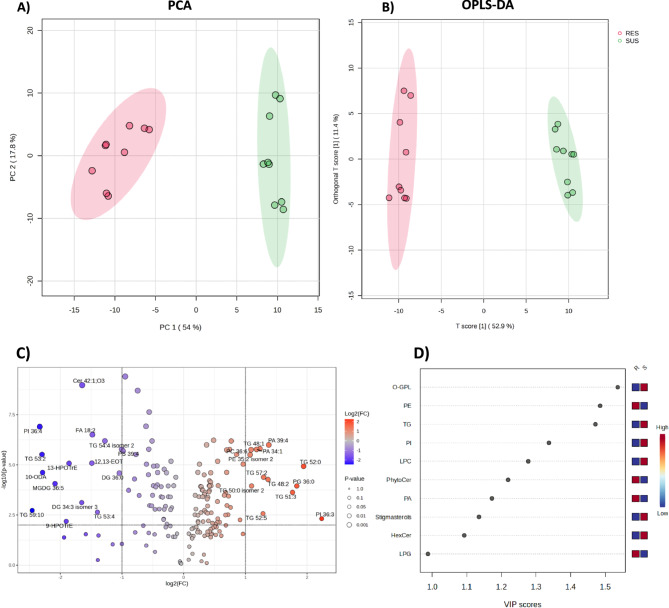
Unsupervised and supervised statistical models of RES and SUS RILs. **A** Principal component analysis (PCA) score plot. **B** Orthogonal Partial Least-Squares Discriminant Analysis (OPLS-DA; R2cum = 0.971, Q2 = 0.964) score plot. In A and B, red and green colours represent bulks of resistant and susceptible RILs, respectively. **C** Volcano plot for annotated features. Red dots are significantly over-accumulated and blue dots are significantly under-accumulated in resistant RILs compared to susceptible ones. **D** VIP plot using chemical classes (FDR < 0.01, top 10 classes) obtained using filtered data. Blue and red marks indicate under- and over-accumulation, respectively. Classes are abbreviated according to the abbreviation list

The dataset was then used to inspect the sample clustering into two groups, resistant (RES) and susceptible (SUS) maize RILs. The unsupervised analysis returned a very clear clustering according to the sample resistance/susceptibility (Fig. 1A). Nonetheless, the supervised model was developed, obtaining a very good discrimination (Fig. 1B), and allowing to point out to the features with highest contribution to the discrimination among the two groups (see Fig. 1C and D).

**Fig. 2:**
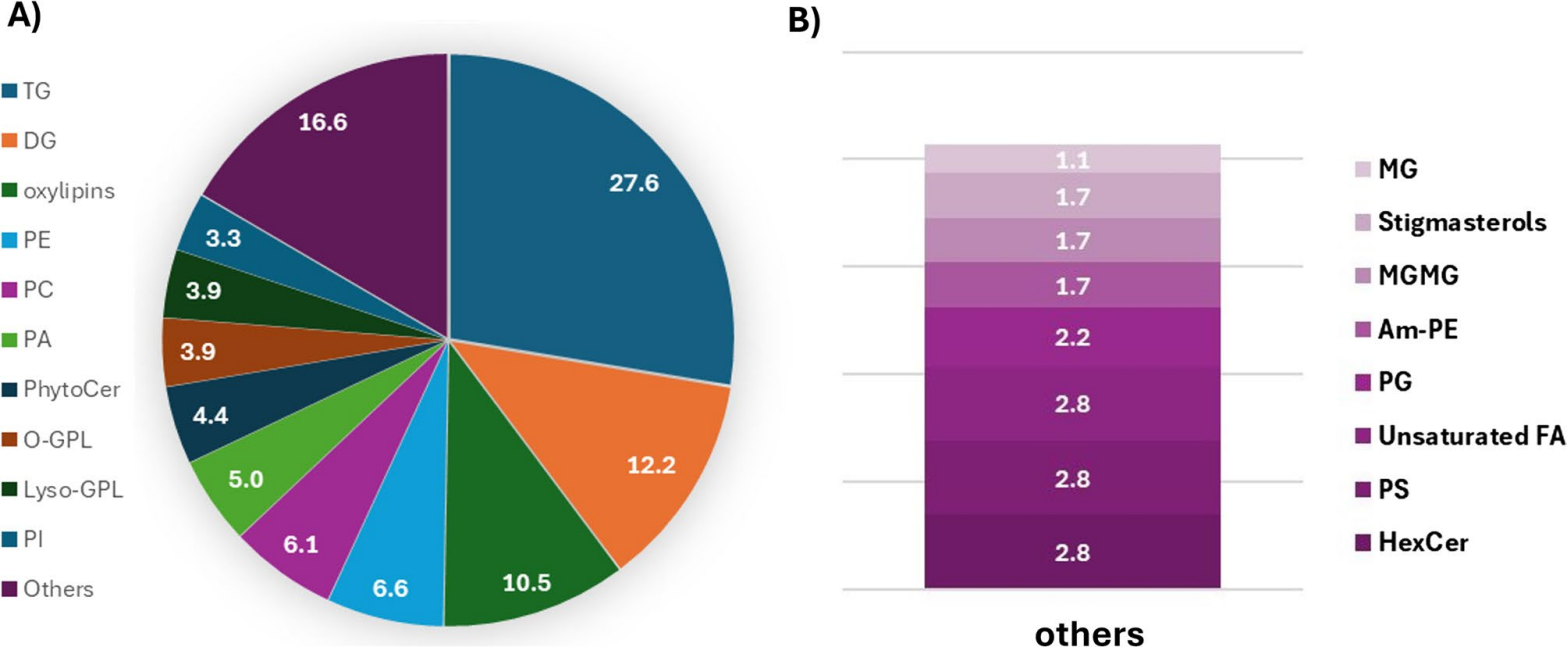
**A** Lipid sub-class pie chart in which the identified metabolites were classified. **B** Distribution of minor classes grouped as “others”. Data are given as a percentage of the total count of annotated compounds. Classes are abbreviated according to the abbreviation list

The annotated metabolites were then classified into biochemical classes and grouped into eleven lipid sub-classes according to the consensus RefMet nomenclature scheme for lipid classification [24], as reported in Fig. 2. Interestingly, besides the expected presence of tri-, di-, and monoacylglycerols (TG, DG, and MG, respectively) as well as glycerophospholipids (GP), compounds belonging to less-represented classes, such as Amadori-glycated phosphoethanolamine (Am-PE), were also detected among the discriminant classes.

**Fig. 3:**
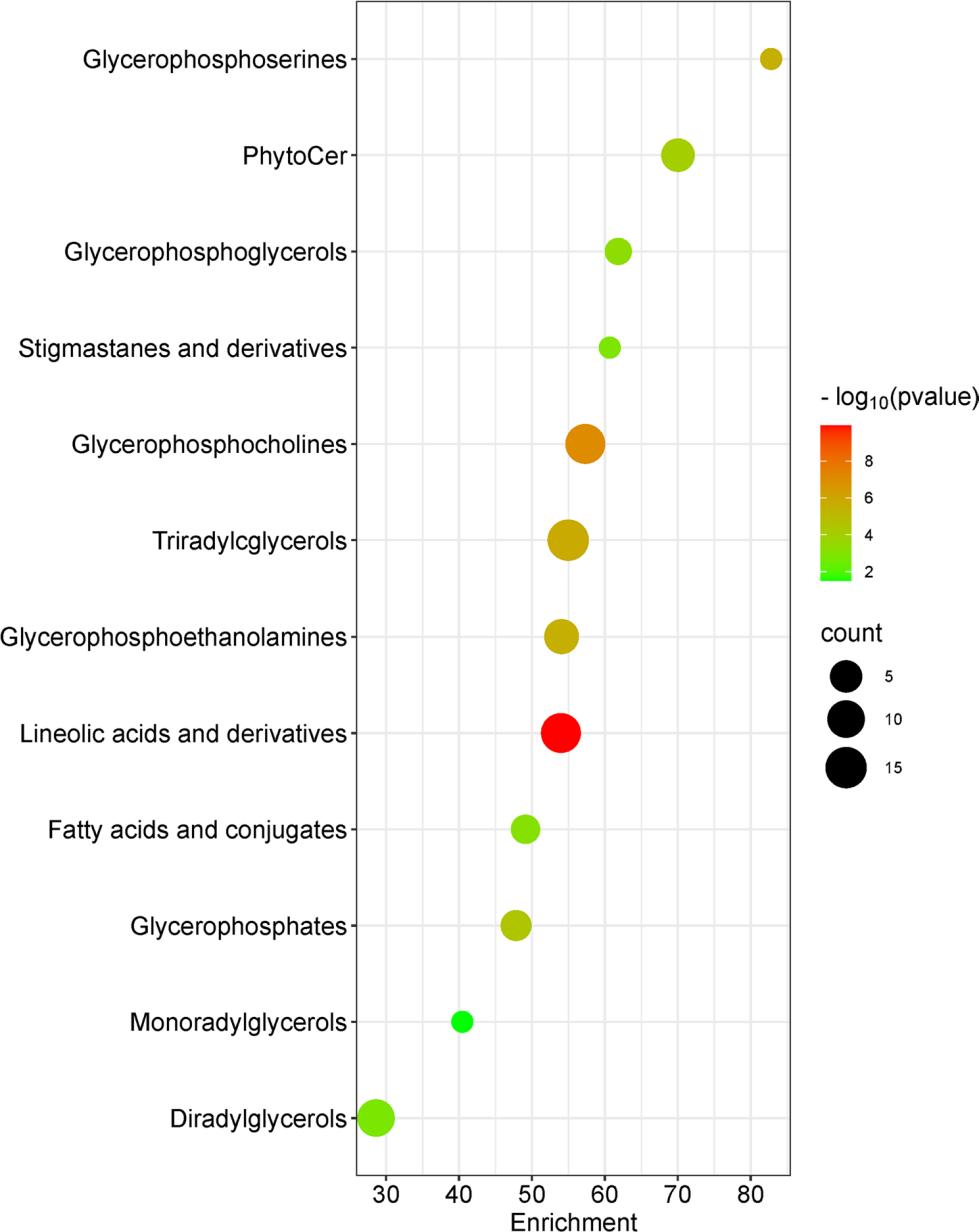
Quantitative Enrichment Dot Plot for the annotated metabolites

In addition, a metabolite Set Enrichment Analysis (MSEA) was performed using MetaboAnalyst 6.0. It is a way to identify biologically meaningful patterns that are significantly enriched in quantitative metabolomic data. MSEA directly investigates if a set of functionally related metabolites without the need to preselect compounds based on some arbitrary cut-off threshold. It has the potential to identify subtle but consistent changes among a group of related compounds. Quantitative Enrichment Analysis (QEA) is reported in Fig. 3 (*p* ≤ 0.01), and showed that compounds mainly involved in the biosynthesis of glycerophospholipids, glycerolipids, and unsaturated FA are enriched among identified metabolites that significantly differ between resistant and susceptible RILs, consistently with what previously reported in the literature [10].

**Fig. 4:**
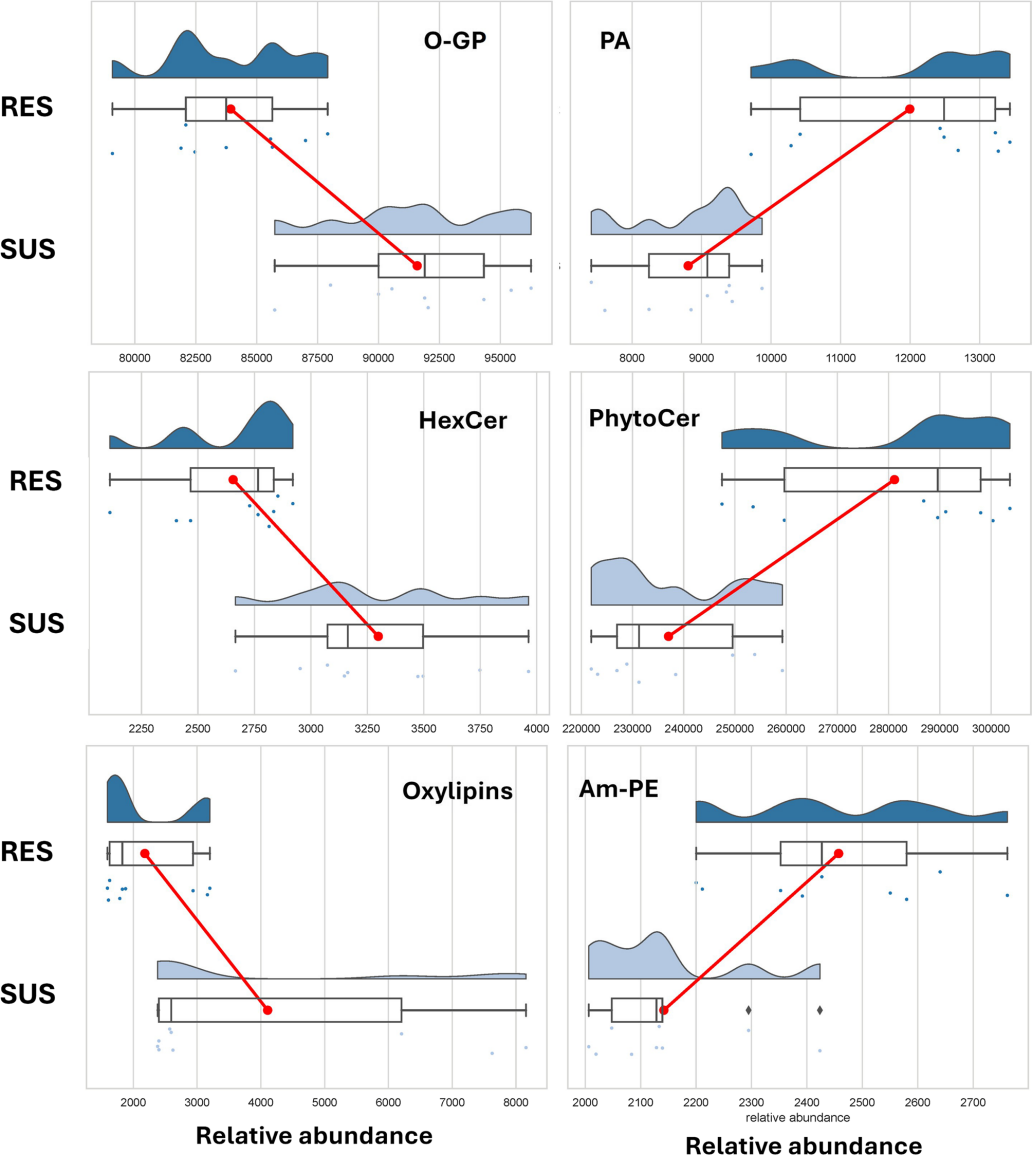
Raincloud Plot representing data distribution expressed as relative abundance for selected classes (data plotted using the SRplot platform). RES: resistant RILs; SUS: suscpetible RILs. Panel **A**: oxidised glycerophospholipids (O-GP); Panel **B**: glycerophosphates (PA); Panel **C**: glycophytoceramides (HexCer); Panel **D**: phytoceramides (PhytoCer); Panel **E**: Oxylipins; Panel **F**: Amadori glycated-phosphoethanolamine (Am-PE)

Our data demonstrated, in agreement with the scientific literature on the topic, that the interaction between the plant and *F. verticillioides* gives rise to a significant modulation of the lipidic profile in maize, and this modulation is different among susceptible and resistant plants. In the current study, several oxylipins have been detected in maize kernels after inoculation with *F. verticillioides*, in agreement with the literature that has shown that lipid peroxidation is enhanced by infection and renders a vast array of oxylipins [8]. Cao et al. [16], using a untargeted approach, showed that one octadecadienoic acid derivative was under-accumulated in resistant compared to susceptible RILs; but with the current approach, focused on lipidomics, more differences for oxylipin accumulation were detected between resistant and susceptible RILs. In particular, 9-oxoODE and 13-oxoODE, the downstream metabolites of the lineoleic acid pathway, as well as methyl-jasmonate and its precursor OPC8:0, were significantly over-accumulated in the bulk of susceptible RILs compared to the bulk of resistant ones (Fig. 4A). Our data agree with those published by Guche et al. [8], who reported on the accumulation of 9-HODE and 10-OPEA, both obtained from 18:2 peroxidation catalysed by 9-LOX enzymes, in one resistant and one moderately resistant line. In particular, the authors observed different kinetics of accumulation between the two inbred lines, with the resistant one reaching a peak at 3 dai and the moderately resistant at 7 dai. A similar pattern was previously discussed by the same group, observing that the fold change of expression between inoculated kernels of one resistant and one susceptible inbred to FER for genes of the LOX pathway became lower than 1 at 14 dai [25]. Therefore, these authors suggested that resistance in maize may depend on an earlier activation of LOX genes and genes for jasmonic acid biosynthesis, as induction was high in the susceptible inbred but delayed. Taken altogether, our data suggested that the front-line signalling cascade following fungal infection was higher in susceptible than resistant RILs at 10 dai, agreeing with the proposed delayed LOX response in susceptible compared to resistant inbreds.

According to previous works [11], the main classes that accumulated more in the bulk of resistant RILs compared to susceptible ones were glycerophosphates (PA) and glycerophosphoethanolamines (PE) (see Fig. 4). These classes are indeed involved in the front-line response to biotic stressors, and commonly show a quick elicitation following a non-specific pathogen attack [9]. On the contrary, oxidised glycerophospholipids (O-GP) are strongly accumulated in susceptible RILs, probably on account of the oxidative events elicited by the pathogen infection.

Interestingly, a differential accumulation was observed in glycophytoceramides (HexCer) and phytoceramides (PhytoCer) (Fig. 4), with the former higher in susceptible and the latter in resistant RILs. Giorni et al. [26] already reported negative correlation coefficients between phytoceramides and kernel infection by *F. verticillioides*.It is indeed reported that FB1 is a potent inhibitor of ceramide synthases (CerSs), able to disrupt sphingolipid metabolism in plants and animals [44]. By inhibition of the CerSs, FB1 has been claimed responsible for the accumulation of long-chain sphingoid bases in plants [44].HexCer are essential components of cell membranes, contributing to membrane integrity and stability during pathogen attack, and they play a key role in the signal transduction pathway that activates defence cascade [27]. In addition, HexCer is also involved in the programmed cell death in infected tissues. Cell death was shown as a chronic toxic effect induced by FB1 in maize seedlings regardless of the genotype susceptibility or resistance to FER in the field, although the acuteness and the kinetics of its chronic phytotoxicity may depend on the maize genetic background [28]. These authors suggested that, from a fungal point of view, cell death caused by FB1 would favour the beginning of the *F. verticillioides* necrotrophic phase and colonization. Therefore, we hypothesize that FB1 occurrence might be the causative agent for phytoceramide differential accumulation in RES and SUS samples.

Am-PE has been detected in both susceptible and resistant RILs. While Am-PE have been extensively described in human plasma as markers of glycation [29], to the best of our knowledge, such compounds are herein reported in maize following *F. verticillioides* inoculation for the first time. According to our data, Am-PE, early glycation products formed when reducing sugars react with phosphatidylcholine lipids, were slightly over-accumulated in resistant compared to susceptible samples (Fig. 4B). While protein glycation is extensively described in plants in the context of stress-elicited modifications [30, 31], studies on lipid glycation are scant, and nothing is known so far about their role in plant resistance towards pathogens. Nonetheless, it has been reported that controlled glycation may be involved in methylglyoxal signalling and regulatory events in plants [31]. Intriguingly, it has been observed in rice that a lower methylglyoxal level confers broad-spectrum resistance against bacterial pathogens [32]. Consistent with this observation, a higher content of Am-PE in resistant RILs might be related to a higher and quicker consumption of methyl glyoxal. Although still very preliminary, this hypothesis surely deserves further exploration.

**Fig. 5:**
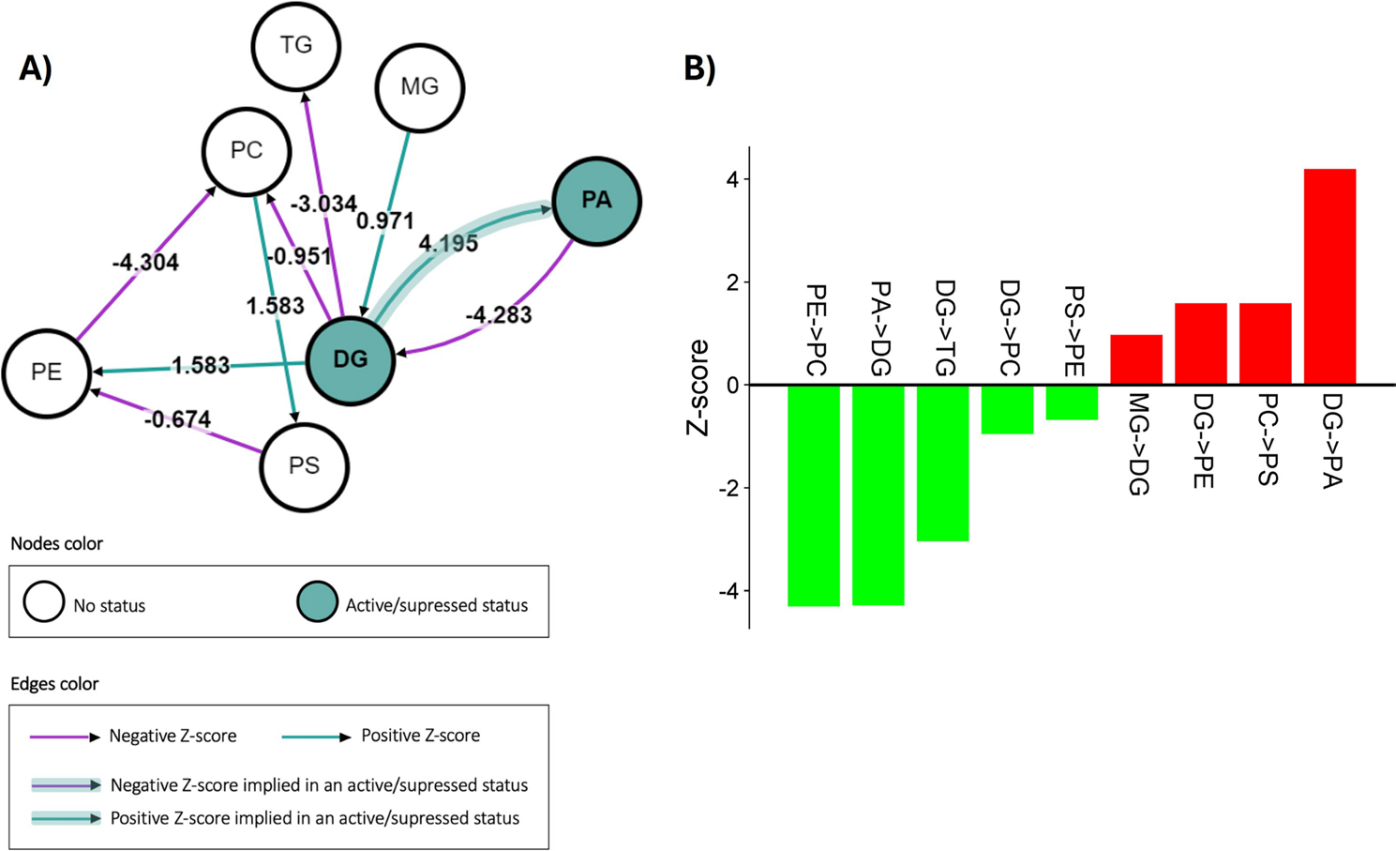
BioPAN pathways analysis. **A** Lipid subclass active pathways. **B** Reaction chains Z-scores (RES versus SUS samples)

Consistent with our previous works [10, 11], the glycerophospholipids metabolism is elicited in resistant lines. Glycerophospholipids have a role in the maintenance of membrane integrity and fluidity as well as in the regulation of the plant defence machinery [33]. Using the Pathway Reaction Analysis tool BioPAN to explore the interconnection among the under-/over-accumulated lipid classes, it was shown that in resistant inbred lines, glycerolipids, mainly DG and PC, are transformed into PA (Fig. 5). The graph reported in Fig. 5A represents the pathways obtained from the annotated compounds (purple arrows for negative Z-score and green arrows for positive Z-score). The nodes correspond to lipid classes, and the directed edges between two nodes symbolise a reaction between these two classes. Green coloured nodes are active pathways, while the colour of the edges depends on the value of the Z-score, green for positive scores and red for negative scores (see Fig. 5B for more details). Although the BioPAN web-based tool was originally developed for mammals [23], it can also be successfully used for common lipid classes across kingdoms, when pathways and functions are conserved, providing a valuable tool to better visualize interactions.

Interestingly, in resistant lines, DG accumulation is favoured over MG/TG and further transformed into PA and PE (Fig. 5). DG itself can act as a signalling molecule, participating in the regulation of protein kinase C and other signalling proteins [34], while the cell membrane component PE can act as a reservoir for other signalling compounds such as N-acetylethanolamines [35]. PS are accumulated in susceptible lines from PE and PC, consistent with their role in signal transduction and programmed cell death [36]. More significantly, the analysis returned a strong PA accumulation in resistant lines compared to susceptible ones, in agreement with their increased resistance towards fungal attack (Fig. 5). PA originates from PC upon phospholipase D hydrolysis, and from DG through the action of diacylglycerol kinase. PA is involved in several defence pathways, among them the activation of mitogen-activated protein kinase cascades, the regulation of hormone signalling pathways, such as those involving abscisic acid and jasmonic acid, as well as in the dynamics of the cytoskeleton, which is important for cellular responses to pathogen attack, including the reinforcement of cell walls and the formation of defence structures [37, 38]. In addition, it has been demonstrated that PA can antagonize H_2_O_2_ and NO in inducing programmed cell death [39].

Among differentially accumulated classes, stigmastanes and derivatives are more abundant in resistant RILs than in susceptible ones, specifically, 16:0-Glc-Stigmasterol and its precursor sitosterol, as well as ricinoleic acid being higher in R versus S samples. According to the literature, stigmasterol has been described as a potential signalling compound for cellular defence, whose content can be modulated by converting it to sterol conjugates such as steryl glucosides [40]. Our data about 16:0-Glc-Stigmasterol accumulation are thus in line with such modulatory activity. Similarly, the higher levels of ricinoleic acid found in resistant RILs are in agreement with several authors describing the antifungal potential activity exerted by ricinoleic acid and other unsaturated fatty acids observed in vitro against pathogens such as *A. niger* and *P. roqueforti* [41–43].

Cao et al. (2022) identified differential genes in RILs. Although a direct statistical comparison between the lipid profiles and the transcript level is not possible due to differences in the methodological set up of the two studies, it should be noted that several annotated genes are potentially related to lipid metabolism pathways. In particular, Zm00001d014820 and Zm00001d046850, overregulated in resistant samples, belong to the GDSL esterase/lipase family, promoting lipid-related defense response in plants following biotic/abiotic stress. Zm00001d038343 encodes for the bidirectional lipid inositolphosphotransferase enzymes, capable of converting PI and ceramide to inositol-phosphorylceramide (IPC) and DAG, and vice versa. Such enzymes may play an important role in modulating plant programmed cell death associated with defense by promoting sphingolipid metabolism and regulating ceramide accumulation. Such relations should be better investigated through dedicated multiomics studies.

As a limitation of this study, it should be pointed out that the extraction method is less specific for more polar lipids and therefore the overall lipid signature could be skewed to more apolar compounds. Nonetheless, this choice has allowed us to compare the results with those already obtained in our previous studies. Considering the difficulties in annotation and the complexity in understanding the overall lipid modulation, adopting comparable and consistent protocols over time is a necessary compromise.

It should also be noted that, under the applied conditions, the experimental design does not allow for understanding whether the shifts in the lipidome are a biological effect directly related to FB1 accumulation or a less specific response to the fungal insult, or even a result of both phenomena. A deeper understanding of the proper biological events involved in the lipid modulation would require a multiomics approach and the use of in vitro testing under very controlled conditions.

It should be noted that understanding the differential lipidomic accumulation between maize resistant and susceptible varieties to *Fusarium verticillioides * can significantly aid breeders in developing Fusarium Ear Rot-resistant varieties. A deeper knowledge of the lipid pathways that are activated in resistant maize, may indeed enable breeders in targeting key pathways in their breeding programs; i.e. information on varieties with higher activation of lipid signaling systems can be used to select resistant genotypes, while the identification of specific lipid metabolites associated with resistance can lead to the development of molecular markers for selection programs.

## Conclusions

Taken altogether and considering the aforementioned limitations, our data demonstrated once again the complex interactions occurring at lipidome levels during plant-pathogen interaction. Besides the involvement of well-described classes such as oxylipins and phospholipids, this study pinpointed the differential accumulation of PhytoCer compared to HexCer as well as of Am-PE in resistant lines. Whether such lipid modulation difference among susceptible and resistant lines is a result of the pathways disruption caused by FB1 accumulation after fungal infection or an inherent response related to the plant genetic background is still to be clarified. Further experiments should be made in this direction under a multiomic approach, also considering the kinetics of fungal infection and FB1 accumulation in plants.

## Supplementary Information

Supplementary Material 1.

Supplementary Material 2.

Supplementary Material 3.

## Data Availability

The Progenesis batch file, alongside comprehensive Excel tables containing 187 annotated compounds in both ESI positive and ESI negative modes, has been uploaded to Zenodo for accessibility and reference: [10.5281/zenodo.14475018](https:/doi.org/10.5281/zenodo.14475018).

## Abbreviations

Am-PE: Amadori glycated-phosphoethanolamine
DG: Diacylglycerols
FA: Fatty acids
FBs: Fumonisins
FER: Fusarium Ear Rot
GP: Glycerophospholipids
HexCer: Glycophytoceramides
Lyso-GP: Lyso glycerophospholipids
MG: Monoacylglycerols
MGMG: Glycosylmonoradylglycerols
O-GP: Oxidised glycerophospholipids
PA: Glycerophosphates
PC: Glycerophosphocholines
PE: Glycerophosphoethanolamines
PG: Glycerophosphoglycerols
PhytoCer: Phytoceramides
PI: Glycerophosphoinositols
PS: Glycerophosphoserines
QEA: Quantitative Enrichment Analysis
RIL: Recombinant inbred line
TG: Triacylglycerols
