# Supplementary material for: Lipids signature shift in Zea Mays L. resistant and susceptible inbred lines in response to Fusarium verticillioides
Source: BMC Plant Biol. 2025 Aug 26;25:1135. doi: 10.1186/s12870-025-07176-4 (PMC12379304; doi:10.1186/s12870-025-07176-4)
Supplement: Supplementary file 3 — Supplementary Material 3. [file 12870_2025_7176_MOESM3_ESM.docx]

**Lipids signature shift in *Zea mays* L. resistant and susceptible inbred lines in response to *Fusarium verticillioides***

Laura Carbonell-Rozas^1#^, Laura Righetti^2^, Noemi Gesteiro^1,3^, Rogelio Santiago^3^, Ana Butrón^3*^, Chiara Dall’Asta^1^

^1^Department of Food and Drug, University of Parma, Parma, Italy

^2^Laboratory of Organic Chemistry, Wageningen University, Wageningen 6708 WE, The Netherlands

^3^Misión Biológica de Galicia, Sede de Pontevedra (CSIC). Pazo de Salcedo. Carballeira 8, 36143 Salcedo, Pontevedra, Spain

**Supplementary Materials**


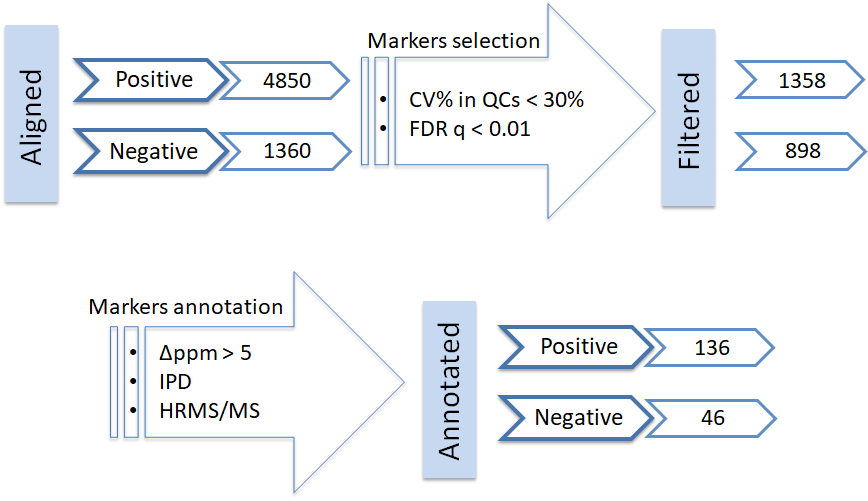


**Figure S1**: Workflow for features selection and identification.


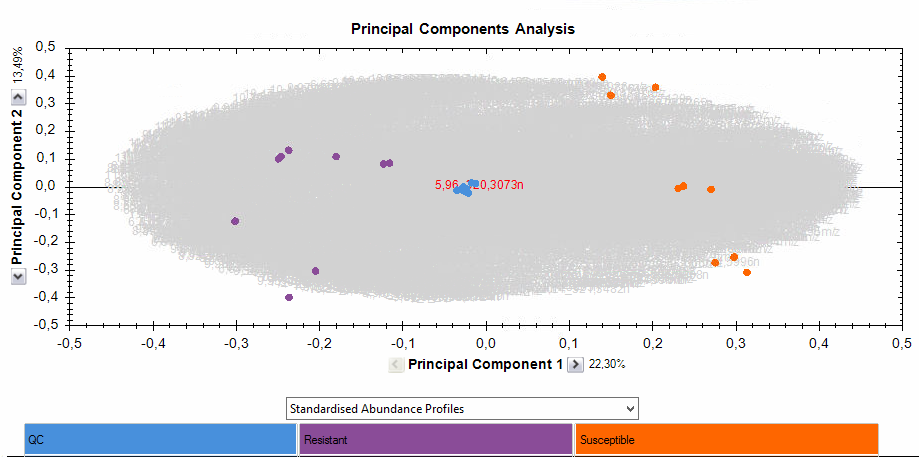


**Figure S2:** PCA score plot obtained for the complete sample set (violet dots: resistant samples; orange dots: susceptible samples; blue dots: QCs). The plot has been obtaine directly from ProgenesisQI using standardised abundance data.
